# Co‐design of the EMBED‐Care Framework as an intervention to enhance shared decision‐making for people affected by dementia and practitioners, comprising holistic assessment, linked with clinical decision support tools: A qualitative study

**DOI:** 10.1111/hex.13987

**Published:** 2024-02-11

**Authors:** Jesutofunmi Aworinde, Catherine J. Evans, Juliet Gillam, Christina Ramsenthaler, Nathan Davies, Clare Ellis‐Smith, EMBED‐Care Programme

**Affiliations:** ^1^ Cicely Saunders Institute of Palliative Care, Policy and Rehabilitation King's College London London UK; ^2^ Sussex Community NHS Foundation Trust Brighton UK; ^3^ Department of Health Sciences Zurich University of Applied Sciences (ZHAW) Winterthur Switzerland; ^4^ Hull York Medical School, Wolfson Palliative Care Research Centre University of Hull Hull England; ^5^ Research Department of Primary Care and Population Health Centre for Aging Population Studies, University College London London UK

**Keywords:** co‐design, decision making, dementia, family caregivers, palliative care

## Abstract

**Introduction:**

Shared decision‐making intends to align care provision with individuals’ preferences. However, the involvement of people living with dementia in decision‐making about their care varies. We aimed to co‐design the EMBED‐Care Framework, to enhance shared decision‐making between people affected by dementia and practitioners.

**Methods:**

A theory and evidence driven co‐design study was conducted, using iterative workshops, informed by a theoretical model of shared decision‐making and the EMBED‐Care Framework (the intervention) for person‐centred holistic palliative dementia care. The intervention incorporates a holistic outcome measure for assessment and review, linked with clinical decision‐support tools to support shared decision‐making. We drew on the Medical Research Council (MRC) guidance for developing and evaluating complex interventions. Participants included people with dementia of any type, current or bereaved family carers and practitioners. We recruited via established dementia groups and research and clinical networks. Data were analysed using reflexive thematic analysis to explore how and when the intervention could enhance communication and shared decision‐making, and the requirements for use, presented as a logic model.

**Results:**

Five co‐design workshops were undertaken with participants comprising people affected by dementia (*n* = 18) and practitioners (*n* = 36). Three themes were generated, comprising: (1) ‘knowing the person and personalisation of care’, involving the person with dementia and/or family carer identifying the needs of the person using a holistic assessment. (2) ‘engaging and considering the perspectives of all involved in decision‐making’ required listening to the person and the family to understand their priorities, and to manage multiple preferences. (3) ‘Training and support activities’ to use the Framework through use of animated videos to convey information, such as to understand the outcome measure used to assess symptoms.

**Conclusions:**

The intervention developed sought to enhance shared decision‐making with individuals affected by dementia and practitioners, through increased shared knowledge of individual priorities and choices for care and treatment. The workshops generated understanding to manage disagreements in determining priorities. Practitioners require face‐to‐face training on the intervention, and on communication to manage sensitive conversations about symptoms, care and treatment with individuals and their family. The findings informed the construction of a logic model to illustrate how the intervention is intended to work.

## 1 INTRODUCTION

Person‐centred dementia care is essential to maximise the quality of care individuals receive, but there are often limited opportunities to engage in decision‐making regarding care and treatment.^1^ Good communication and shared decision‐making are critical components of person‐centred care,^2^, ^3^ with requirements for people living with dementia and their family carers to be involved in decision‐making about clinical care.^4^, ^5^ Too often decisional capacity is overlooked,^6^, ^7^ regardless of whether a person has the ability to make decisions with support.^2^ This means individuals with impaired capacity may have little involvement in decisions about their care and treatment.^7^, ^8^, ^9^

Shared decision‐making is the exchange of information between patients and practitioners (health and social care professionals) about the needs of the patient and the agreement about care and treatment priorities.^10^ Practitioners discuss treatment options with patients (and family when appropriate) and agree on care and treatment decisions. Patients require support to consider their options before making decisions.^11^, ^12^ As dementia progresses, particularly towards the end of life, communication becomes more challenging,^13^ making it difficult to identify and communicate the needs of individuals living with dementia,^14^ and increasing the likelihood of undertreated symptoms and concerns.^15^ Family carers are valuable sources of support for people with dementia, who often rely on them to communicate their needs and make decisions on their behalf.^16^ Decision‐making about care and treatment on behalf of a person with dementia can be stressful. Equally challenging is leaving decision‐making to practitioners.^2^ It is important to have a means of identifying and communicating the symptoms and concerns that people with dementia and their family carers have as the condition progresses, and the plans and priorities for care and treatment.

This study is part of a larger programme called ‘Empowering Better End‐of‐life Dementia Care’ (EMBED‐Care), a programme of research which uses a conceptual framework of integrated palliative dementia care in its approach to co‐design a holistic assessment linked with decision‐support tools (Supporting Information S1).^17^ This study aimed to co‐design the EMBED‐Care Framework, referred to as ‘the intervention’, as an intervention to enhance shared decision‐making, for people living with dementia at home, family carers and practitioners to identify and agree on the priorities of care and how to manage them.

The study objectives were: (1) To co‐design a person‐centred dementia care intervention to enhance shared decision‐making between people affected by dementia, and health and social care practitioners. (2) To construct a conceptual logic model considering context, implementation and mechanisms of impact of shared decision‐making.

## 2 MATERIALS AND METHODS

### 2.1 Design

A series of theory and evidence driven co‐design workshops, using an iterative approach and informed by O'Cathain et al's method of co‐design was used to develop the intervention as a complex intervention for shared decision‐making for individuals affected by dementia.^18^, ^19^, ^20^, ^21^ Co‐design facilitates engagement with end‐users and stakeholders^18^ and has been used in health research to develop, test and refine complex interventions, including for people living with dementia and their family carers.^18^, ^22^, ^23^, ^24^ This study drew on the Medical Research Council (MRC) framework for developing and evaluating complex interventions.^25^, ^26^

### 2.2 The intervention

The intervention was developed from evidence synthesis on integrated palliative dementia care.^17^ The intervention comprises holistic assessment of needs (referring to symptoms and concerns in this article) and review, supported by a person‐centred outcome measure, the Integrated Palliative care Outcome Scale (IPOS‐Dem), linked with clinical decision support tools to support and manage care decisions.^17^ ‘Needs’ refer to symptoms and concerns of the person with dementia.^27^ The IPOS‐Dem was developed from the well‐established, validated, and reliable Palliative care Outcome Scale^28^, ^29^ and the most recent version, the Integrated Palliative Outcome Scale (IPOS).^30^ IPOS‐Dem is person‐centred and assesses multiple health domains, including physical, psychosocial, and spiritual needs of individuals living with dementia. The IPOS‐Dem also asks about the family carer's needs. It begins with open questions of the person and their family's main concerns in the past week.^31^, ^32^ There are three versions of the IPOS‐Dem: a self‐report, and proxy reported versions for family carers and practitioners, respectively. The clinical decision support tools, referred to as ‘rules of thumb’, use best available evidence, presented in simple flow diagrams to support assessment and management of common needs that cause distress in dementia and inform decision‐making about care and treatment.^22^, ^33^, ^34^

### 2.3 Underpinning theoretical model

The co‐design workshops were theoretically driven by a shared decision‐making model called ‘the enriched model of collaborative deliberation in dementia care network’.^35^ The model specifies seven elements to shared decision‐making, including (1) ‘constructive network engagement’ (e.g., involving an individual and their family in decision‐making), (2) ‘recognising the need for a decision now’, (3) ‘defining what to decide on’, (4) ‘developing alternatives’, (5) ‘constructing preferences through deliberation and trying out’, (6) ‘multiple preferences integration’, and (7) ‘evaluating decision‐making’.

This theoretical model of decision‐making informed an initial systematic review on using person‐centred outcome measures to support shared decision‐making in dementia care.^36^ A conceptual logic model, detailing how the intervention worked to improve outcomes and areas of uncertainty, was constructed from the review findings.^25^, ^37^ The logic model detailed how a person‐centred outcome measure could facilitate understanding of an individual's needs by the person, their family carers and/or practitioners completing the measure to identify unmet symptoms or concerns, and discuss and agree priorities for care and treatment. We used the logic model to highlight gaps, including how to manage multiple preferences and priorities for care and treatment, training and support for family carers, and how and when to evaluate the decisions made.^36^ The logic model was used to design the workshops, including the topic areas considered, tasks undertaken and priorities for discussion to address uncertainties.

### 2.4 Participant eligibility

The participant groups were people living with dementia, current or bereaved family carers of people with dementia, and practitioners providing care to people living with dementia in community settings (primary care, community care or social care).

**Eligibility criteria:**

Person with dementia:

- Diagnosis of dementia of any type and severity
- Capacity to provide written informed consent
- Able to speak in English

Family carer:

- Current or bereaved family carer (including close friends) with lived experience of caring for a person with dementia
- Able to provide written informed consent
- Aged 18 and above
- Able to speak in English

Practitioners:

- Any health or social care practitioner providing care for people living with dementia in community settings (e.g., dementia care specialists, community nurses, palliative care specialists, etc.)
- Able to speak in English
- Able to provide written informed consent

### 2.5 Participant identification and recruitment

People living with dementia were identified and recruited through the Dementia Engagement and Empowerment Project (DEEP) group. DEEP is a UK‐based network of people living with dementia that connect people and ensure the views of people living with dementia are represented.^38^ Capacity was assessed through discussion of the research study with experienced dementia researchers who drew on the Mental Capacity Act^39^ to determine the capacity to provide informed consent to participate in the study. Only those with the capacity to consent were recruited to the study. Family carers were identified and recruited from public involvement groups. These groups included the EMBED‐Care Patient and Public Involvement (PPI) Study Reference Panel and the National Institute of Health and Care Research (NIHR) Join Dementia Research (a national online platform for people living with dementia and family carers to register interest in dementia research participation).

Practitioners were identified and approached via the research team's clinical networks, and by contacting home care organisations and NHS community teams identified through the Care Quality Commission website (an independent regulator of health and social care in England and ensures services are accountable to high‐quality care standards).^40^ We searched the Care Quality Commission website for services providing care to people living with dementia at home, such as home care agencies, targeting services in London. Services were contacted by email by the research team to explore their interest in participation. We then used snowballing methods to further identify and recruit additional practitioners with relevant expertise.

All participants received an information sheet about the study via email for the virtual workshops to inform them of what their involvement would include and their right to withdraw. Participants were sent a consent form via email to sign electronically after expressing interest in participation. We received informed consent in person for the in‐person workshops and participants received an electronic copy of their consent form. All workshops had multiple scheduled breaks to reduce fatigue. All workshop attendees were offered a £20 voucher after each workshop to thank them for participating. Practitioners received a certificate of attendance, of which the first two workshops were accredited by the Royal College of Physicians for Continuing Professional Development points.

### 2.6 Patient and public involvement and engagement

Four meetings were held with Patient and Public Involvement and Engagement (PPIE) groups between May 2021 and May 2022. The PPIE groups included people living with dementia, bereaved and current family carers, and practitioners. PPIE members informed the development of this study from study design through to data analysis and interpretation. Members informed the topic guide for the co‐design workshops (e.g. to explore who uses the outcome measure for assessment), and tested, and gave feedback on the intervention prototype for shared decision‐making. Supporting Information S2 details the characteristics of the PPIE groups, their contributions and impact on the research, reported using the Guidance for Reporting Involvement of Patients and the Public (GRIPP2).^41^

### 2.7 Format of the co‐design workshops

Participants completed a demographic form to provide data on characteristics prior the first workshop. A facilitator (J. A., J. G., or N. D.) coordinated each workshop, supported by a scribe (a research assistant) to take notes during each workshop. ND is an experienced facilitator for co‐design workshops.^33^, ^42^ J. A. and J. G. attended training on conducting co‐design workshops prior facilitating the workshops. We created and used vignettes (Supporting Information S3) about using the intervention for shared decision‐making and used Google Jamboard (a digital whiteboard with sticky notes) to aid discussions (Supporting Information S4 and S5). The virtual sticky notes were used to enhance the discussion, by asking participants to elaborate on their thoughts and ideas. Each workshop ran for up to Two hours. The workshops were audio recorded using Zoom recording and transcription functions, or a digital recorder. Participants were not asked any confidential information over Zoom and researchers ensured that the discussion remained centred on development of the intervention. Audio recordings were transcribed using the Microsoft Word transcribe function and manually checked and amended for accuracy.

**Figure 1:**
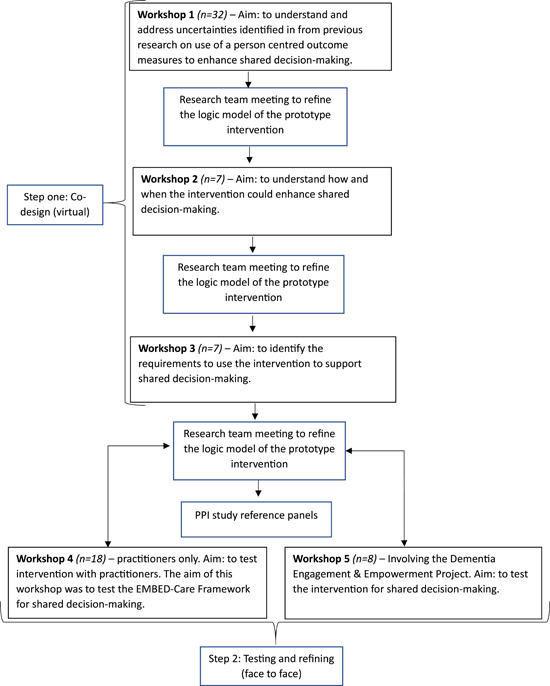
Overview of the workshops to co‐design and refine the prototype intervention for shared decision‐making for people living with dementia.

Five iterative co‐design workshops were undertaken between June 2021 and October 2022 to construct and test a prototype of the intervention for shared decision‐making within the development of the intervention (Figure 1). Each preceding workshop informed the subsequent workshops to build understanding and develop the intervention iteratively. A summary of the discussions from the preceding workshop was presented at the start of the subsequent workshop detailing how it informed the current workshop and the focus of the current workshop. The research team met to discuss and refine the intervention after each workshop. The workshops involved two steps:

1. Step 1 was to co‐design the Framework as an intervention for shared decision‐making (workshops 1–3). These workshops sought to address areas of uncertainty identified in the systematic review,^36^ and priority areas identified by the EMBED‐Care PPI Study Reference Panel to develop the intervention for shared decision‐making.
2. Step 2 was testing and refining the intervention (workshops 4–5). These involved asking people living with dementia, family carers and practitioners to test the Framework as an intervention and provide feedback on any changes required to ensure it could be used for its intended purpose in routine care.

### 2.8 Data analysis

Scribe notes were analysed between workshops to determine the main discussion points on using the intervention to enhance shared decision‐making. Data analysis used Reflexive thematic analysis,^43^ with data managed and analysed in NVIVO 12. Analysis was undertaken by J. A, who has experience in qualitative research. Initial codes and themes were discussed and pursued with CE and CES, both experienced qualitative researcher with clinical backgrounds in palliative care and dementia. The final themes were reviewed by the research team (N. D., C. E. S., C. E. and J. G.), all experienced in qualitative data analysis.

Coding began with initial coding after reading and data familiarisation.^43^ Data coded for patterns on how the intervention is used to enhance shared decision‐making, and the requirements to use it in clinical routine care. The codes were refined and used to construct initial themes. The underpinning logic model and shared decision‐making model informed and guided the initial codes, before theme development.^36^ This initial deductive analysis explored the data for patterns on how and when the intervention could enable shared decision‐making, including frequency of use, who was involved in using it and how. The analysis also explored approaches to manage multiple preferences for care and treatment and what was required to use it by the person with dementia, their family carers and practitioners. We then inductively analysed the data for patterns on using the intervention for shared decision‐making and the potential benefits of its use. Once the data had been coded, they were refined into themes. The results of the workshops refined the intervention and logic model of the intervention for shared decision‐making.

### 2.9 Ethical approval

This study was reviewed and approved by the Health Research Authority and NHS Research Ethics Committee, REC Ref: 20/LO/0295 (London Queen Square Committee).

## 3 RESULTS

### 3.1 Co‐design workshops and participant characteristics

**Figure 2:**
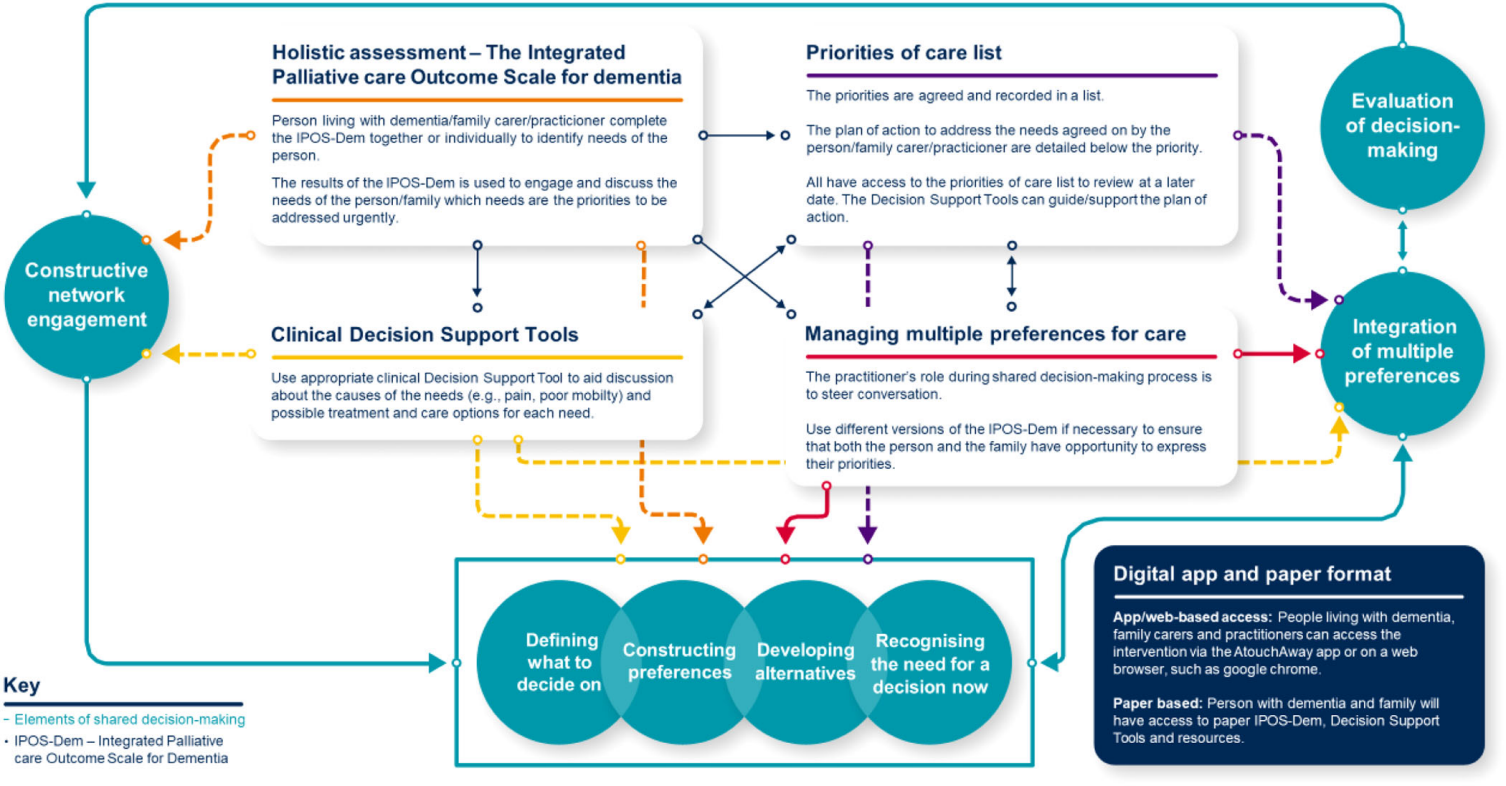
Intervention diagram, detailing how individual intervention component interacts with different elements of shared decision‐making.

Five workshops were undertaken involving 54 participants (Table 1). Three workshops were held virtually from June 2021 to November 2021 to construct the logic model and prototype intervention (Figure 2) for shared decision‐making, and two follow‐up workshops were held in person from September to October 2022 to test the prototype intervention (Figure 1). The participants included people living with dementia, family carers (*n* = 18) and practitioners (*n* = 36) (paid practitioners providing care to people living with dementia), reflecting a multidisciplinary team from specialist palliative care, community and home care, such as nurses, and palliative care specialist (Table 1). The DEEP group were recruited as a group and seven group members attended the workshop, three of whom were individuals living with dementia.

**Table 1:** Participant characteristics.

	Family carers (*n* = 18)	Practitioners (*n* = 36)
	*n*	*n*
Age: Median (range)	63.5 (42–80)	43.5 (28–63)
Gender (women)	16	32
Marital status		
Single, never married/in civil partnership	4	6
Cohabiting, married or in a civil partnership	12	29
Other	4	0
Missing	0	1
Ethnicity**		
White (English/Welsh/Scottish/Northern Irish)	18	26
White other (German, Irish, South African and Portuguese)	0	4
Black (African, Caribbean, or other)	0	1
Asian (Pakistani, Sri Lankan or other)	0	1
Not reported	0	4
Level of education		
Degree or equivalent	10	28
GCSE/GCE level	5	5
Other qualifications	3	1
Missing	0	2
Practitioner role^a^		
Registered nurse or nursing assistant		20
Home care provider		7
Other (including primary, palliative care, and dementia care practitioners)		8
Missing	0	1

GCE, GCSE, general certificate of education; general certificate of secondary education; GP, general practitioner.

a ‘Other’ included: palliative medicine consultant, dementia project support officer, improvement manager, advanced clinical practitioner, hospice education lead, GP, care home manager, Patient and Public Involvement coordinator, and field support supervisor.

### 3.2 Findings from the reflexive thematic analysis

Three themes were generated from across all the co‐design workshops on how and when the intervention could be used as an intervention to enhance shared decision‐making for people with dementia, their family carers and practitioners, and what was required to enable use and change practice. The themes comprised ‘knowing the person and personalisation of care’, ‘engaging and considering the perspectives of all involved in decision‐making’, and ‘training and support activities’.

### 3.3 Theme 1: Knowing the person and personalisation of care

The intervention can allow for the personalisation of care by enhancing understanding of the needs of the person with dementia across health domains. Specifically, the IPOS‐Dem could be completed with individuals whilst assessing their needs or after practitioners have completed their assessment involving the person and their family carer. Using the IPOS‐Dem should involve the person (if able), their family carers and practitioners to strengthen understanding on ‘knowing the person’. Whilst the IPOS‐Dem can support identification of individual needs to inform treatment and care plans, this requires negotiation and alignment with individual cultural preferences and religious beliefs. These characteristics impacted the willingness to engage and discuss care needs.

We had a new client come on board from social services… so I wanted to talk to him about how this might pan out and where he wants to stay and things, and so he said ‘Allah will decide and not a moment sooner’, and that was it. End the conversation… [Workshop 2—Home care provider manager]

I think you also have to think about cultural things as well about having those conversations and about where that sits in particular, family setups or the relationship, like if we're talking about a spouse or a child of someone where those things, because they can have massive implications on even having the discussion. [Workshop 1—Dementia Clinical Nurse Specialist, community care]

Flexibility in using the intervention is key to personalisation of care and impacting shared decision‐making. For example, if suspected that the needs of the person with dementia changed, the IPOS‐Dem could be completed to assess the changes in the needs of the person with dementia, identify unmet needs and review management. Participants felt that the intervention should be used at least monthly and more frequently if required to tailor and ensure personalisation of care, and review the outcomes of care and treatment to meet identified priorities.

Like I was saying, if you just standardise everything [in reference to the language used within the intervention], you take away the whole aspect of being person centred… what's appropriate and would be standard for me wouldn't be for [mentioned name of another participant]. [Workshop 5—Person with young onset dementia]

Participants felt that some symptoms should be reported solely by the person with dementia (when able) ahead of decisions for care and treatment. For example, people living with dementia reporting of their pain symptom needed to be considered over proxy reporting as only individuals can truly express the pain they are experiencing. The anticipated benefit of using the intervention, specifically the IPOS‐Dem, was empowerment of individuals and family carers to understand and communicate needs and priorities for care, and improved confidence to participate actively in decision‐making about care and treatment.

Yeah, I think one of the advantages of having a tool like this [referring to the intervention] and for family carers to be able to understand all the different parts of it and how it works, it gives them strength to argue their case more. [Workshop 3—Bereaved family carer for uncle]

### 3.4 Theme 2: Engaging and considering the perspectives of all involved in decision‐making

Using the intervention for shared decision‐making requires consideration of the perspectives of all involved in caring for the person with dementia. Practitioners were required to be sensitive to the different needs raised by the person and their family carer to allow all involved to be engaged, and this needs to be reflected in the approach to using the Intervention.

Well, everybody knows that dementia is an individual thing to each individual, but I do feel that home carer, with the relative, the husband, wife, or whoever, is probably the best placed to give a positive and accurate view of the patient, the person living with dementia*…* [Workshop 1—bereaved family carer for husband]

Practitioners had the opportunity to explore in depth with the person and their family carers in situations where there were disagreements about the symptoms and priorities for care. The use of accessible language was necessary to allow the person and their family carer to engage in the decision‐making process. Using different versions of the IPOS‐Dem (self‐report and proxy versions reported by family carer) aided discussion and empowered the person and the family carers to feel confident to communicate what was important to them and contribute to informed decisions about treatment and care.

…but I suppose use it [referring to the IPOS‐Dem] as an opportunity to have a discussion about using the tool to discuss what that difference is about and maybe try and understand why his wife is scoring differently to him. [Workshop 1—Dementia Clinical Nurse Specialist, home care]

It was important for family carers to feel that they were heard as this supports their confidence and engagement in discussions about the care of the person with dementia. The role of the practitioner during the discussion about priorities and care decisions was to steer the conversation and ensure that the person with dementia and family carer had opportunities to express what was important to them and that all had opportunity to contribute towards the discussion and the decisions made.

I also think that we are so protective as a family… I live with my family and there are certain things I will not tell them, even if I am in pain, so, if I was to fill that in [in reference to the IPOS‐Dem], that I am in pain and if they were to fill that, no. How are we going to get the true answer on there? What are we going to do? Because so far we've had the professionals where they talk to your partner and I wasn't asked about anything, and that's just not on. [Workshop 5—Person with dementia]

### 3.5 Theme 3: Training and support activities

The intervention was seen by practitioners as a way to build knowledge about the needs of the person with dementia and inform decisions about care. However, practitioners expressed concerns about using the intervention on an electronic device (e.g., a tablet) because it required practitioners to look away from the person. This acted as a potential barrier to the interaction between the person with dementia and practitioner, thus impacting the identification of needs. A paper version of the IPOS‐Dem was identified as a potential solution to retain patient‐practitioner interaction as people with dementia may be more familiar with this style of clinical visit, as opposed to the use of a tablet. Practitioners required flexibility with how the intervention worked. For example, some felt they would use IPOS‐Dem as part of a conversation with the person with dementia, whilst others would ask the person or their family carers to complete the IPOS‐Dem in the first instance.

We have iPads with systems. We did trial it sometime [referring to using iPad to conduct assessment with patients], but at certain points the patients really don't want to engage with me because I'm looking at the iPad, so it's just much more difficult to do it, so I gave up and just take the hard copies and talk with them through it. It's because they need the eye contact…it's much more difficult to try and engage with the person, but I think the hard copy in there can help a little bit… [Workshop 4—Nurse, NHS community team]

People living with dementia and family carers require support to understand how the intervention could be used to enhance shared decision‐making. Information about how to use intervention, such as when to use it, needs to be provided in ‘small chunks’. Animated videos were identified as an information aid. Participants recommended short, animated video on the use of the intervention to facilitate shared decision‐making. The video needs to provide the relevant information quickly to help understand how to use the intervention for shared decision‐making. Some family carers felt it helpful to receive training on using the clinical decision support tools for different IPOS‐Dem symptoms, such as constipation as this understanding could allow them to be more engaged in discussing the needs and priorities of the person, and making decisions.

…because animated videos as well can get the message over and sometimes people can get concerned when they see other people, but the animation can really sort of have an effect [Workshop 3—Bereaved family carer for mother]

I think if for all our ones [in reference to people seen in their homes] who sort of aren't so used with the technology side, it's also not overwhelming the information and keep it very simple [Workshop 3—home care provider]

Face‐to‐face training for practitioners was seen as fundamental to understand how the intervention works, and listening and communication skills training to ensure that people living with dementia and family carers perspectives were considered. Communication training was deemed important to manage sensitive conversations and reduce concern for people with dementia and their family carer. In addition to training, sufficient time with the intervention before implementation was required to increase familiarity and confidence to use it in clinical practice. Familiarity with the intervention was essential to support the interaction with the person with dementia and their family carer, and not act as a barrier.

This is new so give me a couple of weeks to use it. I think it's very messy at the minute. I feel a bit unprofessional sitting in a patient's home, scrolling through all these notifications and not knowing where to click, I can't find things properly, things disappear. [Workshop 4—Nurse, NHS community team]

Promoting the anticipated benefits was essential to implementing the intervention to facilitate shared decision‐making. For practitioners, anticipated benefits relating to skills development and having a sense of fulfilment from optimal care provision for people living with dementia was required. For family carers, they predicted that the intervention would improve communication and involvement in discussions about care.

You'll learn so much about your client, but so much about yourself, if you're up for it, if you don't just want to do a job. You actually want to be on the journey that [mentioned name of practitioner 2] was referring to, and we say that to our clients as well. You know, we are joining you on a journey here. [Workshop 3—Home care agency manager].

## 4 DISCUSSION

We co‐designed a complex intervention for shared decision‐making for people affected by dementia. The findings constructed a conceptual logic model detailing what was required for use, when to use it, and how the intervention worked, considering implementation, mechanisms of impact and outcomes.

A key finding from this study was that the intervention could be used to build knowledge of an individual's needs and personalise their care through agreeing on priorities and goals of care. This requires the person and/or their family carer to complete the IPOS‐Dem, and use the clinical decision support tools to participate in care and treatment decisions. This is the ‘constructive network engagement’ component of Groen Van de Ven's enriched model of collaborative deliberation in dementia care networks.^35^ This requires knowing and involving the individuals who will contribute to identifying the needs of the person with dementia and making decisions, including the individuals themselves. Our results resonate with existing literature on the importance of involving the person with dementia in decisions about their care and treatment,^44^, ^45^, ^46^ which allows for the care received to align with the person's preferences.^12^ People living with dementia may rely on family carers for support to make decisions on their behalf when they are unable to. Although decision‐making can be challenging for family carers, they often do not feel comfortable leaving decision‐making power to practitioners.^47^ The use of the intervention by people affected by dementia and practitioners could empower people affected by dementia to feel confident in communicating their needs, preferences and priorities about their care and treatment. Use of the IPOS‐Dem in particular could facilitate communication, through completing and discussing the identification of symptoms and concerns.

Based on the findings and discussion with the research team, we developed a ‘priorities of care list’. A ‘priorities of care list’ intends to provide people affected by dementia an opportunity to express and agree a list of priority symptoms to be addressed, and the plans to manage them. The ‘priorities of care list’ compliments the IPOS‐Dem assessment and clinical decision support tools as it requires, after discussing the needs of the person with dementia, to record the main priorities and plans to address them. For example, if pain is the priority, this is detailed, and the plan to manage pain could be to review medication.

A vital requirement to use the intervention was training and support for all involved. Previous studies highlight that training and support for family carers to use an internet‐based intervention on caring for a person with dementia can improve understanding of the needs of the person and how to provide care.^48^, ^49^, ^50^ The findings informed the development of instructional, animated videos. Previous studies on the use of videos as decision support tools have been found helpful in understanding and communicating information, and engaging people with dementia.^51^, ^52^ A telephone and email helpline were created to support family carers and practitioners to use the intervention and provide ongoing support to implement the intervention. We have developed a detailed manual using simple language, on different components of the intervention, such as how to use it to support shared decision‐making. An in‐depth, in person training for using the intervention for shared decision‐making has been developed for all practitioners. A ‘train the trainer’ (or champion) approach was decided on as an approach to implement the intervention for shared decision‐making to support knowledge development for staff and encourage use.

A component of shared decision‐making in dementia care is managing multiple preferences and disagreements in priorities.^35^ In this study, participants indicated that disagreements could serve as an opportunity for the person and their family to express what their concerns are and for these to be discussed and considered in the decision‐making process. In some instances, separate discussions with the person with dementia or their family carer, respectively, could be appropriate to provide opportunities for individuals to speak freely about perceived needs, without causing distress to the person or their carer. Using self‐report and family proxy report versions of the IPOS‐Dem (self‐report and/or proxy report) could allow the person and family carers to highlight the needs of the person with dementia from the respective perspectives, facilitated by the practitioner to understand and discuss the differences in concerns and priorities.

Within the training for practitioners, we incorporated strategies to manage multiple priorities and preferences for care and treatment. These included the use of different versions of the IPOS‐Dem as appropriate to elicit information about the needs of the person with dementia and for practitioners to be sensitive and skilled in their communication to take account of different views in the decisions made.

### 4.1 The logic model of the intervention for shared decision‐making

**Figure 3:**
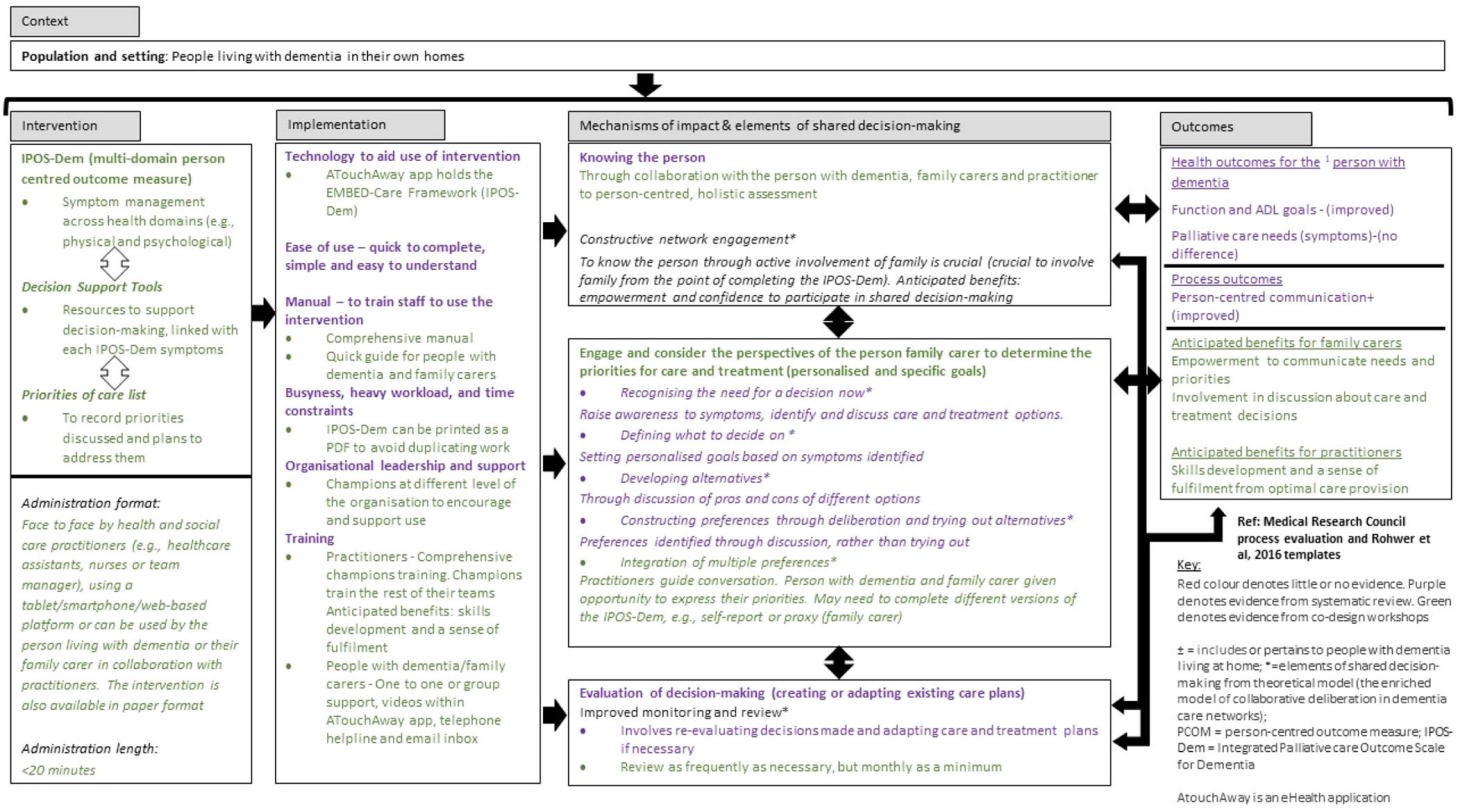
A logic model of the intervention for shared decision‐making for people affected by dementia and practitioners.^37^

The results of the co‐design workshops were used to refine the logic model of the intervention for shared decision‐making developed originally from the systematic review.^36^ The logic model details the components of the intervention, the intervention requirements for implementation and linkages between mechanisms of impact and anticipated benefits (Figure 3). A key part of using the intervention for shared decision‐making is the support activities for people affected by dementia and practitioners (see implementation box in Figure 3). This aligns with the literature on the importance of skill, education and training for practitioners^53^, ^54^ to enhance knowledge^55^, ^56^ and confidence in providing end of life care.^57^ The logic model highlights areas of uncertainty in using the intervention, such as a priorities of care list (Figure 3). The processes around the priorities of care list requires further research to understand how it impacts on shared decision‐making and care outcomes.

### 4.2 Strengths and limitations

Due to COVID‐19, three of the five workshops were conducted virtually. This enabled recruitment of participants from across the UK, allowing us to develop the intervention with the perspectives of individuals representing many different services across the United Kingdom. Co‐design approaches require a balance of power between all researchers and participants, with equal decision‐making power.^18^ In this study, the researchers were facilitators and prompted participants during discussion. This was necessary to ensure that the topic of conversation remained relevant to the intervention for shared decision‐making, although participants were not stopped from raising ideas and leading conversations about using the intervention for shared decision‐making. Participants were also encouraged to raise ideas and lead the discussion about using the intervention for shared decision. PPIE were also represented in developing and testing the intervention for shared decision‐making to ensure that it is relevant for people living with dementia and those involved in their care (detailed in Supporting Information S2).

We used multiple methods to identify and approach practitioners to encompass the diversity of disciplines, teams and services caring for people with dementia in the community. We used existing networks and broadened through contacting services through the Care Quality Commission website to identify practitioners with little or no research experience. We acknowledge that our recruitment approach via email through existing networks may have excluded individuals with limited digital access or literacy. The majority of participants identified as White British. Participants from diverse ethnic backgrounds would provide a richer understanding of the cultural congruence of the intervention, and opportunities to strengthen for respective ethnic groups. The decision‐making process for individuals from ethnically diverse backgrounds may differ from that of western society. Culture and religion are essential to consider as they contribute to understanding who an individual is. Our priority in a near future feasibility study is the involvement of people from diverse backgrounds to understand how the intervention could enhance shared decision‐making for them.

### 4.3 Implications for clinical practice

Shared decision‐making is an important aspect of person‐centred care, and essential to good care provision for people living with dementia.^4^ The intervention for shared decision‐making has been developed to be used by individuals with dementia, family carers and practitioners alike to support decision‐making. The IPOS‐Dem, in particular, could empower individuals and their families to be confident in discussing symptoms and concerns and be involved in the decisions to address them. Practitioners can use the intervention for monitoring and ensuring that care delivery aligns with individual priorities, which could positively impact care outcomes. There is evidence of the benefits and values of using person‐centred outcome measures, on outcomes in palliative care, such as facilitating decision‐making, symptom identification, communication and monitoring about an individual's symptoms.^58^, ^59^, ^60^, ^61^, ^62^

### 4.4 Future research

Future research should explore how the intervention can enhance shared decision‐making for individuals from diverse backgrounds to refine the intervention, ensuring inclusivity. Future research will undertake a process evaluation and feasibility study of the intervention for shared decision‐making for people living with dementia at home. We will explore the processes by which the intervention enhances shared decision‐making and impacts care and quality of life outcomes.

## 5 CONCLUSION

Using co‐design, we developed a theoretically driven intervention for shared decision‐making for people living with dementia. The intervention requires contributions from the individual (when able) and/or their family carer to understand an individual's needs and to agree priorities for care and treatment. The workshops highlighted how the intervention could enhance the knowledge of the person, facilitating in turn personalised care. The workshops highlighted understanding on managing disagreements on priorities between the person and family carer. People with dementia and family carers require support to understand how to use the intervention, particularly, how to complete and interpret the IPOS‐Dem. Practitioners require face to face training on intervention and on communication to manage sensitive conversations with the person and their family carer.

## AUTHOR CONTRIBUTIONS

**Jesutofunmi Aworinde**: Conceptualisation; investigation; writing—original draft; methodology; validation; visualisation; writing—review and editing; software; formal analysis; project administration; data curation. **Catherine J. Evans**: Conceptualisation; funding acquisition; writing—review and editing; visualisation; methodology; formal analysis; supervision. **Juliet Gillam**: Writing—review and editing; visualisation. **Christina Ramsenthaler**: Writing—review and editing; visualisation. **Nathan Davies**: Writing—review and editing; visualisation. **Clare Ellis‐Smith**: Conceptualisation; writing—review and editing; visualisation; methodology; formal analysis; supervision.

## CONFLICT OF INTEREST STATEMENT

The authors declare no conflict of interest.

## Supporting information

Supporting Information.

Supporting Information.

Supporting Information.

Supporting Information.

Supporting information.

## Data Availability

The data that support the findings of this study are available on request from the corresponding author. The data are not publicly available due to privacy or ethical restrictions.
