# Supplementary material for: Co‐design of the EMBED‐Care Framework as an intervention to enhance shared decision‐making for people affected by dementia and practitioners, comprising holistic assessment, linked with clinical decision support tools: A qualitative study
Source: Health Expect. 2024 Feb 11;27(1):e13987. doi: 10.1111/hex.13987 (PMC10859658; doi:10.1111/hex.13987)
Supplement: Supplementary file 2 — Supporting Information. [file HEX-27-e13987-s004.DOC]

Guidance for Reporting Involvement of Patient and the Public (GRIPP).^1^

| Aim | The aim of patient, public involvement, and engagement (PPIE) was develop an intervention – the Empowering Better End of Life Dementia Care (EMBED-Care) Framework for shared decision-making and to ensure that was to ensure that it was relevant and useful to people living with dementia, their family carers and health and social care professionals. |
| --- | --- |
| Methods | Three groups of PPIE groups met on three occasions between May 2021 and October 2022. The groups were the EMBED-Care study reference group and the JA’s PhD PPI study reference panel. This group was established to inform the use of the EMBED-Care Framework for shared decision-making. This group is made up of 5 members, an individual living with dementia, a current and former family carers, and practitioners working in an NHS community team and home care agency. The individual living with dementia is met individually to allow opportunity to fully contribute their ideas. The second group is the wider EMBED-Care PPIE group, comprising 6 people living with dementia and former family carers. The third group is the Dementia Engagement and Empowerment Project (DEEP) group, comprising 7 people living with dementia and former family carers. DEEP is a UK-based network of people living with dementia that connect people and ensure the views of people living with dementia are represented. The PPIE groups were asked their thoughts on using the different components of the EMBED-Care Framework for shared decision-making, such as the language used within the intervention (e.g., was the term ‘training’ appropriate with family carers?). The groups were asked to consider using the EMBED-Care Framework for shared decision-making (e.g., how much involvement should the person with dementia/family carer have when using the Framework, and how to manage multiple preferences and priorities for care) and what would be helpful to use it. Once a prototype of the Framework for shared decision-making was developed the groups were asked to review the Framework and the manuals developed to support its use. |
| Study results | The PPIE groups contributed significantly to the development of the EMBED-Care Framework for shared decision-making. Their involvement impacted on:   1. Initial discussions in the co-design workshops on how to manage multiple preferences during the shared decision-making process. 2. The EMBED-Care Framework. 3. Language used within the EMBED-Care manual on support for family carer (i.e. support, not training). 4. How the EMBED-Care framework is used for family carers, but using it and providing feedback on the appearance of the EMBED-Care Framework and ease of use. 5. The comprehension of EMBED-Care manuals generally, and specifically for shared decision-making. 6. The final EMBED-Care Framework resources, such as videos explaining information about the intervention. Their feedback has led to changes on the animated video, e.g. such as what the appearance of the characters. |
| Discussion and conclusions | PPIE have been involved from the inception of the EMBED-Care Framework for shared decision-making. The contributions of the PPIE group have led to changes on the EMBED-Care Framework and the resources developed to support its use as described above. However, there were suggested changes from the PPIE members that are not possible ahead of the next stage, which is to use the EMBED-Care Framework in clinical routine care. These changes include changes to the appearance of the documents of the EMBED-Care Framework and to the language on the audio recording accompanying the support videos. These requested changes have been document and will be implemented after the feasibility study of the EMBED-Care Framework. |
| Reflections | PPIE contributions to the EMBED-Care Framework as an intervention for shared decision-making is crucial to ensuring that it remains relevant to people living with dementia, their family carers and practitioners involved in their care. We anticipate the intervention will support shared decision-making and empower people living with dementia and their family carers to feel confident to engage in care decisions with practitioners. |

1. Staniszewska S, Brett J, Simera I, et al. GRIPP2 reporting checklists: tools to improve reporting of patient and public involvement in research. *bmj*. 2017;358
