# Supplementary material for: Co‐design of the EMBED‐Care Framework as an intervention to enhance shared decision‐making for people affected by dementia and practitioners, comprising holistic assessment, linked with clinical decision support tools: A qualitative study
Source: Health Expect. 2024 Feb 11;27(1):e13987. doi: 10.1111/hex.13987 (PMC10859658; doi:10.1111/hex.13987)
Supplement: Supplementary file 3 — Supporting Information. [file HEX-27-e13987-s005.docx]

# Case 1:

Mr Daniels is a 79-year-old who lives at home with his daughter, his primary carer. He has a diagnosis of Alzheimer’s dementia. He has recently been experiencing pain in his left leg, low mood, and poor appetite. When a community nurse visits Mr Daniels at home, Mr Daniels says that he is fine and keeping well. The nurse speaks with Mr Daniel’s daughter, who gives a different account saying her father seems quite low in mood speaking less and less, seems to have pain in his leg when walking, and is not eating as much. The nurse asks Mr Daniels if he has any pain. Mr Daniels then mentions the pain in his leg, when asked again how he is feeling says, he feels unhappy, but is unsure why.

**Please use the EMBED-Care Framework.**

# Case 2:

Ms Boateng is a widow who lives alone. She has no children and has lost contact with all her family after moving to the UK from Ghana over 40 years ago. She was diagnosed with mild Alzheimer’s dementia after being admitted to the hospital following a urinary tract infection. She recently developed breathing difficulties. Following discharge, she was referred to a PCN team. Her neighbour visits her every day to check in on her. Carers from a local care agency visit twice a day to support her with dressing, preparing meals, and shopping.

**Please use the EMBED-Care Framework.**

# Case 3:

Mr Cox is a 70-year older man with a diagnosis of moderate dementia and appears to currently struggles to verbally communicate. He lives with his wife. He has been experiencing issues with sleeping at night recently and falls asleep at random times during the day. He also has difficulties with his mobility.

**Please use the EMBED-Care Framework.**
