# Supplementary material for: Co‐design of the EMBED‐Care Framework as an intervention to enhance shared decision‐making for people affected by dementia and practitioners, comprising holistic assessment, linked with clinical decision support tools: A qualitative study
Source: Health Expect. 2024 Feb 11;27(1):e13987. doi: 10.1111/hex.13987 (PMC10859658; doi:10.1111/hex.13987)
Supplement: Supplementary file 4 — Supporting Information. [file HEX-27-e13987-s003.pdf]

How do we use the IPOS-Dem

Completing the IPOS-Dem - Person with dementia and family carer (including friends)

Person with dementia and their family carer complete the IPOS-Dem together

↓

Person with dementia, their family carer and a nominated practitioner together discuss the symptoms and concerns that have been identified by the IPOS-Dem, symptoms and concerns that are priorities, discuss the options available for those symptoms and concerns

↓

Person with dementia, their family carer and practitioner agree goals of care, i.e. the plan of action to address the symptoms and concerns. Decisions are guided by a decision aid

Practitioner to agree with person with dementia and family when the goals of care will be reviewed, e.g., in 2 weeks, 4 weeks, or as and when needed (e.g., when the person or family carer feels there has been a change)

1. Who completes the assessment and when? such as on a monthly basis as default, but needs to be flexible

IPOS-Dem could be a useful tool to trigger discussions. But not sure how often I'd get it out. At the end of life, often the family carer involved

IPOS-Dem something used when people ready to discuss things, and involve people with decisions able to contribute to.

To be used whenever people feel comfortable/ready to discuss. Could be useful at diagnosis

Useful tool to prepare family carers for symptoms/situations that may occur later on

Family carer involved in the conversation - review throughout end of life

A conversation guider for when people are ready for this conversation about end of life. Document can be referred back to for family members.

IPOS-Dem completed at initial assessment when referred to a service, like Home Care. Then, at points of regular review - monthly, and if things change for the person with dementia and/or the carer

hours? The decisions made are determined by the resources available and the practitioners delivering the care. IPOS-Dem could help to work across teams - with many carers coming in and out. Get a sense of how things have changed

Completing the assessment - the 1st option is the best one. If need to involve a practitioner it will take a very long time to complete

This could enable people receiving a new diagnosis to consider what they may want as the condition progresses. It would also then enable family members to consult this at a later stage.

This is a great way for the family carer to keep an eye on things. This is helpful for the family to monitor and see trends, and change and communicate this

2. Which practitioner is best suited to liaise with? such as a GP/ community nurse

I can see community nurses, matrons would want to use this. But I wonder how much weight it would really hold to make decisions when it came down to it.

practitioners involved. IPOS-Dem needs to be part of the journey to be used by different practitioners. There is not one practitioner - we had a nurse coordinator who visited 6 monthly, and 9-10 different people at different stages. Most important was

As family carers you come in with no knowledge about dementia. The training package is very important. As dementia progresses you learn about dementia and providing care

Ensuring that people living with dementia remain at the heart of the process. Ensuring this is a living document, and remains current and accessible.

3. What are the challenges you expect with working in this way? consider leaderhsip of organisation, busy workload, having a champion, etc

For family carer what do think the barriers would be for you to use the IPOS-Dem and decision support aid?

If this could be a shared document, it would enable whichever health practitioner who is working currently, most closely to lead at any given time. Always ensuring the person themselves is at the heart of this.

Challenges - written down about what clients wanted at the end of life, but sometimes the care plan is overlooked at the end of life. Having things written down essential

Things (symptoms) can change very quickly and a very different decision might need to be made in the evening than would have been made in the morning

Recognition of symptoms might not be easy with no training

Lack of monitoring for people with dementia. Healthcare practitioners don't see the changes

Would it really be referred to by all practitioners. What happens if we are not there? The person is not always kept at the centre

How do we managing multiple priorities

Managing different priorities and preferences for care

- Try to understand the different preference and reasons, i.e., from person and family's view
- Temporarily agree on a decision with views to review at next appointment

How is multiple preferences and care for treatment managed in your experience?

Family carer recognise needs, that may be the person with dementia doesn't recognise

How can we ensure everyone can participate the process. Going from pain, weakness, lack of energy - is it too much at one time. Using pain scales helpful to understand not just pain - yes or no. Think about arrange of issues

Difficulty of family carer when using the tool being able to be balanced - very hard as a family member

Can be conflict between what family know the PWD would have felt was important and their current urgent needs

Ask the family carer what we can bring to support them. Ask the person how they are. Using IPOS-dem would be an extension of this. Role of the practitioner help to steer the conversation, IPOS-dem could help this

Preserving dignity of PWD is a challenge - if person does not recognise needs that a family member or carer has observed (e.g. pain) would not want to contradict them, but still want to address they symptom

Sometimes how a person is managed at home comes down to very practical things - like money, £250 for a night sitter

Taking someone to the doctor for a problem you've been observing over many weeks. But when see the doctor - person doesn't remember the problem and denies for example that been in pain

How do we use the clinical decision-aid

Clinical decision-aid

Use appropriate clinical decision aid to support decisions around care, e.g., decision aid on emotional wellbeing of the person with dementia

Who gets the decision aid? The practitioner/ family /person with dementia or all?

Is it helpful to have access to all the decision aids at all times, or only when the IPOS-Dem is completed?

Task 1 - The EMBED-Care app/paper format

EMBED-Care app and paper format

- **App**: People with dementia and practitioners will be provided with tablet with an app. Family carers will have option to download an app to access the IPOS-Dem, linked in with resources, such as the decision support. Goals of care will be recorded here.
- **Paper based:** Person with dementia and family will have access to paper IPOS-Dem and decision aid

How do you envisage this will work if paper based?

Would you expect that goals of care (decisions made together regarding care) to be recorded in the app as well as in the care plans or just in care plans?

Mentoring - NIHR has a mentoring scheme - could be helpful for family carers and easy to dip in and out of at own pace

Could be a really useful tool for family carers and help to consolidate/reduce the number of assessment tools being used, save time.

Training - bite-size, small chunks of 15-30 minutes

What we still don't know:

What should training include to enable family carer to use the IPOS-Dem e.g. [illegible] IPOS-[illegible] on shared decision-[illegible]

Family carers can be relied upon to be very good observers. We kept a diary, such as sleep pattern. Decision plan hasn't got anything about diet and hydration, that's very important for family carers to understand

Electronic records means the family members are being kept out of reporting and assessment, and review because they have no access to the records

system called open pass- where family members are able to log on digitally to read all the care notes written by the carer who has been in which means they can access it remotely as well if they are unable to go in and see their [illegible]

Most family carers can be relied upon to be very very good observers - need to utilise these skills. Needs to be an expansion of the decision support areas in the IPOS-Dem. Nothing about nutrition and hydration support?

Important not to overwhelm families with too much information, especially at early on post diagnosis. Could offer a very simple explanation of the tool to let families know it exists and they can go back to it when appropriate.

[illegible] guide can be very useful even before training. Getting people onboard at the start - possibly introductory training before formalised. Might be difficult for family carers to do weekly, needs to be simple and not too [illegible]

[illegible] be[illegible]ers to s[illegible]

[illegible] Mindful that these are difficult topics for family and PWD. Mindful of the language used, needs to be inclusive and to avoid certain terms (e.g. wandering). Consult tools/materials put together by PWD on [illegible]

[illegible] around the new tool. Training around communication around significant points in people[illegible] lives. Ensure lan[illegible] used is inclusive[illegible] the person with dementia - 'wandering' use[illegible] IPOS-Dem is a l[illegible]

Helpful to have information on what are the palliative care services available in the local area - Hospice@home, Marie Curie night sitters etc..

Listening skills and recognition that families really do know what they're talking about and have valuable contributions to make.

Training - Listening skills, interpersonal relationships to adapt style to the person with dementia, the family carer so we can actively listen to the person and family.

Could a drop in be useful? Either on line, zoom or in person - for people living with dementia, family and professionals

'Bitesize' training is key - family carers are often stressed and very busy with competing responsibilities. Training needs to be small chunks that work for them.

Task 2

What we still don't know:

How culture impacts on decision-making

From your experience, how has culture played a role in decision-making for care and treatment of people with dementia

Recognising different and diverse backgrounds. Personal beliefs will influence decisions (or openness to discussing/making decisions)

Mindful of generational/cultural gender roles. Responsibilities of husbands and wives.

Using language that is acceptable to the PWD and family (around death and dying etc.)

Cultural beliefs -'Alha will decide what will happen, and not a moment sooner', advance care planning may be less of a priority for the person as not seen as within their gift

Sometimes PWD with ESL can lose ability to speak English, need to accommodate/enable people to communicate in first language

Fear of being 'put away' - where does that fear come from? Personal past experience/environment earlier in life.

What we still don't know:

What additional resources would be helpful to have, e.g.,

links to websites like alzheimer's society for the person with dementia and family

Manuals explaining how and when to use the IPOS-Dem and decision support aid

Anything else?
