# Supplementary material for: Co‐design of the EMBED‐Care Framework as an intervention to enhance shared decision‐making for people affected by dementia and practitioners, comprising holistic assessment, linked with clinical decision support tools: A qualitative study
Source: Health Expect. 2024 Feb 11;27(1):e13987. doi: 10.1111/hex.13987 (PMC10859658; doi:10.1111/hex.13987)
Supplement: Supplementary file 5 — Supporting information. [file HEX-27-e13987-s001.pdf]

# Training content for health and social care professionals

| Training sessions | Content |
|---|---|
| Session 1: IPOS-Dem, clinical decision resources, and EMBED-Care app | 1. Presentation on the rationale and intention IPOS-Dem, clinical decision resources, and EMBED-Care app.<br>2. Health and social care professionals will interact with the app, accessing and completing the IPOS-Dem, looking through the clinical decision resources, setting alert threshold |
| Session 2: Shared decision-making in dementia care, communication and listening skills, role of culture, language considerations | 1. A combination of interactive session and discussion of case studies/vignettes to help consider shared decision-making, communication, listening, sensitivity to person with dementia individual context |

Are the planned activities sufficient and engaging? if not, how can this be improved? How long should the sessions be?

When doing through form tried to divide it up as to whether it is an examination/observation/observation over time. Many observations over time.

Are you aware of any material/resources to help train practitioners in communication/listening skills

Dementia end of life care module - a lot of this based on case studies and discussing options to get used to communicating.

People acting as patient and carer - helped to develop listening and communication skills.

Primary care teams and care home staff doing the course at the same time. Certain people allocated roles - role play with case study to follow

We have adopted a 'light' coaching approach with our clients. We teach 'active listening' and 'effective questioning' skills in the context of dementia care which helps communication.

We also teach DISC personality profiling tools with all staff which is great to understand different 'types' and approaches that may be needed when communicating

An opportunity to have a former carer or someone like that? Their input can be important. No representation may not be the right way forward.

Therefore family members appropriate to assess patient alongside healthcare professional

# Coaching for family carers

| Coaching video | Content |
|---|---|
| 1. IPOS-Dem (5 mins)<br>2. Clinical decision resources (5 mins)<br>3. EMBED-Care app (5 mins) | A coaching video describing how the IPOS-Dem is used, e.g. explaining some of the terms the IPOS-Dem uses.<br>Other coaching videos will include how to use the clinical decision resources/rules of thumb and the EMBED-Care app. |
| One page guide | As well as video coaching, we will have a one page guide for the IPOS-Dem and clinical decision resources |

In Kent through dementia strategic improvement group - care coordinators. They could be the ones in Kent that pick it up. Admiral nurses could also have a role in this. Caseloads however still too big. When to have the conversations?

Is current plan sufficie[illegible]else would you like to see in coaching video[illegible]d be helpful[illegible] about having a FAQ s[illegible]the app?

Problem with dementia care - it is always somebody else's business not who you happen to approach.

Needs to say early on - 'getting on board with this, will mean this to your loved one'. An example of how it can changes things. The outcomes need to be simply put in human terms from the start.

Case studies can be very powerful. Having this or something similar at the start can attract people towards something and realise how it may help them. Get them on-board at the start.

For those who ar[illegible] not used to tech [illegible] need the info to [illegible] simple and not overwhelming. People like simpl[illegible] pictures with few words. Not just written text.

Joint dementia research - one of the things that have been successful is animated videos. They can get the message over. Animation can have an effect.

A face-to-face discussion/presentatio[illegible]n might be needed for some families to accompany a short leaflet (where they cannot use internet or other technology)

Block providers will not have the space to have those conversations. Those on lower incomes who do not have private care may not have access to this.

Who will find time to sit down with the carers? Home care providers will not be able to - it will not happen. Holistic and supportive conversations will no[illegible] happen. May do with private care packages

Problem with dementia care - so fragmented. Prep work often falls on social services or voluntary sector services. Where need to aim some of the education.

Talk about the tool at the same points that you talk about end of life (which can happen at any time).

Information session - run by researchers or health and social care practitioners?

Outcomes - e.g. more will understand how your relative expresses themselves, or pain management. Not to talk about joined up working but things that will matter to individuals and their families

In addition to video coaching, we plan to have one page guide. Helpful to send a package that gives instructions on how to use the App beforehand?

What do you think of the term coaching instead of training?

"Give (someone) instructions as to what to do or say in a particular situation"

Maybe 'supporting' family carers? Some may be reactionary to something like that. 'Support with family carers'. Not all will want to understand training/coaching but will want support

Introduce elements of 'coaching' to the workshop. A collaborative process with family carers. Talking about workshops may be good enough.

'Empowering family carers' - more generic and then say there is this training, this workshop, this coaching element.

Some may find the word coaching too 2021 (especially if they are more elderly it may put them off). Or they may feel patronized - although empowering may do the same thing.

**Workshop for family carers or Support are better terms**

**'Support workshop'**

New tool which may help - but at the same time want your input. Two-way process inviting people's input to improve and to learn from carers. 'Demonstration' as a possibility

Workshop - implies you will have to do work but that we will work on it together. Like that term. Implies you will get something to take away and what will help them day-to-day

It is a two-way process. Thinking about pain management - and the family can train the professional about how the individual responds to pain

Useful when first introducing it. If make it too complicated it will have a negative effect on family carers and they will feel under pressure

**We have many spouses who care and they want things to be as easy as possible**

Paper guide would be helpful to have something they can continuously refer back to

May have many who are not very technology-minded and won't use tablets or iphones

# Outcomes for family carers

What outcomes are important to consider for family carers as a result of using the EMBED-Care intervention to enhance shared decision-making? e.g. carer burden

REASSURANCE is the key outcome - to help a person understand and anticipate changes

Avoid the word 'outcome'. Instead, what difference will it make it to you and your quality of life

That this tool will support a TAILORED end of life for that person; really responding to a person's wishes

Some people may not understand outcomes - that needs to be a different term.

the tool will support a 'What Matters Most?' end of life for each individual person; the tool will help a 'Good Death'

One of the outcomes - people will know that they are being listened to.

Instead of carer burden I think of it as a journey with various stop off points.

Feeling heard. Outcomes from the tool seem to be looking at 'if this happens, do this next'. Can get the support that they need, sooner.

'What matters most' needs to be somewhere in this. It isn't just one size fits all in what end of life care looks like.

By being involved it will mean that there are no surprises. It is about equipping family carers about what is to come.

Make sure to use phrases that all can understand and relate to.

Dislike the term 'carer-burden'. Implies that looking after your relative is a burden. But it is actually that they don't get the right help, and right support when they need it. That is the frustration.

With tool - may be able to show e.g. how pain has increased. Otherwise, a nurse e.g. who does not know the patient can make a decision single-handedly. Shared decision-making.

Gives family carers strength to argue their case more. Can put information in front of the professionals and it gives them some authority

Make sure outcomes are in language that family carers want to hear. We are good at talking in NHS and social care speak. Family carers need to be involved in the language.

Family carers to be involved throughout

Supporting DIGNITY at end of life

Had to learn how best to communicate with individuals, it may be that loved one respond a certain way when in pain for example.

Access to services and getting them all to communicate with each other properly.

Need a more family-friendly way of saying shared decision-making.

Treat family carers as part of the team and understand the value they have.

Ester approach/Ester principle - Swedish approach which has been going for decades. Tries to sort out fragmentation of support. Adopted in Kent and Medway

Some will not understand 'shared decision-making' either

Look at it in terms of what benefits are important for the family? E.g. better communication, better decision-making.

Supporting you with your life and where it is now as a carer rather than the 'burden'. Burden is used far too often when it comes to elderly people.

Completing the assessment together, all have had a discussion around care. All together coming to an agreement about what is best and the approach to take. Then reviewing it

# Outcomes for health services

## What outcomes are important to consider for services as a result of using the EMBED-Care intervention to enhance shared decision-making?

care improvement works website - put out key lines of enquiry that CQC uses. Look at this and will be able to find where being involved in this may fit for providers

Our outcomes will be the client outcomes. We want the family, and the person to have the best end of life in their own home or transition to another place.

Advantage of using this - more holistic approach. Often healthcare professionals are rushing in and looking at one problem rather than the person.

OUR Outcome is the same as the Client/Family Carer

Scale up the leaders and the care assistants within organisation to have these conversations and to have more joined up working. Empower and add to learning for their professionals

Caring, joint working, leadership, safe, responsive - it probably falls under many of them (CQC key lines of enquiry)

Tool should be flagged and highlighted as a way to help providers be even better and to get wonderful outcomes for families which matters most to us.

More holistic - more likely to get access to the right services at the right time.

Providing staff with skills - communication, listening skills

Getting quicker support - more joined up working as a result of using the tool

Tool will map to outstanding provider as trying to do all in best possible way and use all that is there.

Outcomes that any healthcare team is that the person and their family is at the centre of all.

If it means there is a greater understanding of the person and their needs at the moment - leads into targeted support by teams in place to do so.
